# Peripheral Blood TCRβ Repertoire, IL15, IL2 and Soluble Ligands for NKG2D Activating Receptor Predict Efficacy of Immune Checkpoint Inhibitors in Lung Cancer

**DOI:** 10.3390/cancers16162798

**Published:** 2024-08-08

**Authors:** Andrea Sesma, Julian Pardo, Dolores Isla, Eva M. Gálvez, Marta Gascón-Ruiz, Luis Martínez-Lostao, Alba Moratiel, J. Ramón Paño-Pardo, Elisa Quílez, Irene Torres-Ramón, Alfonso Yubero, María Zapata-García, María Pilar Domingo, Patricia Esteban, Rebeca Sanz Pamplona, Rodrigo Lastra, Ariel Ramírez-Labrada

**Affiliations:** 1 Medical Oncology Department, University Hospital Lozano Blesa, 50009 Zaragoza, Spain; disla@salud.aragon.es (D.I.); mgasconr@salud.aragon.es (M.G.-R.); amoratielp@salud.aragon.es (A.M.); equilezb@salud.aragon.es (E.Q.); irentorresr@salud.aragon.es (I.T.-R.); ayuberoe@salud.aragon.es (A.Y.); mzapatag@salud.aragon.es (M.Z.-G.); rlastrad@salud.aragon.es (R.L.); 2 Aragon Health Research Institute (IIS Aragón), 50009 Zaragoza, Spain; pardojim@unizar.es (J.P.); lmartinezlos@salud.aragon.es (L.M.-L.); jrpanno@salud.aragon.es (J.R.P.-P.); pesteban@iisaragon.es (P.E.); rebecasanz@iconcologia.net (R.S.P.); 3 CIBER de Enfermedades Infecciosas (CIBERINFEC), 28029 Madrid, Spain; eva@icb.csic.es; 4 Microbiology, Radiology, Pediatry and Public Health Department Medicine, University of Zaragoza, 50009 Zaragoza, Spain; 5 Instituto de Carboquímica (ICB-CSIC), Miguel Luesma 4, 50018 Zaragoza, Spain; 6 Department of Microbiology, Pediatrics, Radiology and Public Health, University of Zaragoza, 50009 Zaragoza, Spain; 7 Aragon Nanoscience Institute, 50018 Zaragoza, Spain; 8 Aragon Materials Science Institute, 50009 Zaragoza, Spain; 9 Immunology Department, University Hospital Lozano Blesa, 50009 Zaragoza, Spain; mpdomingo@icb.csic.es; 10 Infectious Disease Department, University Hospital Lozano Blesa, 50009 Zaragoza, Spain

**Keywords:** lung cancer, immune checkpoint inhibitors, predictive biomarker, T cell receptor repertoire, cytokines

## Abstract

**Simple Summary:**

The development of immune checkpoint inhibitors has revolutionized the treatment of lung cancer by becoming the standard therapy for advanced non-small cell lung cancer that lacks specific genetic mutations. However, not all patients respond equally, underscoring the need for biomarkers to predict treatment response. To address this, a study was conducted with 55 lung cancer patients treated with immune checkpoint inhibitors to investigate whether biomarkers like TCRβ diversity and certain cytokines linked to T cell activity could predict the response to immunotherapy. While higher TCRβ clonality and specific cytokine levels appeared to be associated with improved survival rates, the findings were not statistically significant. Specifically, higher levels of IL-2 and IL-15 were linked to shorter overall survival, with high IL-15 levels increasing the risk of death threefold in multivariable analysis. Although further research with larger sample sizes is needed for confirmation, these results offer promising insights into potential markers for predicting responses to immune checkpoint inhibitors.

**Abstract:**

The development of immune checkpoint inhibitors (ICIs) has changed the therapeutic paradigm of lung cancer (LC), becoming the standard of treatment for previously untreated advanced non-small cell lung cancer (NSCLC) without actionable mutations. It has allowed the achievement of durable responses and resulted in significant survival benefits. However, not all patients respond; hence, molecular biomarkers are needed to help us predict which patients will respond. With this objective, a prospective observational study was designed, including a cohort of 55 patients with NSCLC who received ICIs. We studied whether biomarkers such as TCRβ and specific cytokines involved in the regulation of T cell activity were related to the immunotherapy response. In the survival analysis, it was found that patients with higher TCRβ clonality, lower TCRβ evenness, higher TCRβ Shannon diversity and lower TCRβ convergence had higher overall survival (OS) and progression-free survival (PFS). However, no statistically significant association was observed. Regarding cytokines, those patients with higher levels of IL-2 and IL-15 presented statistically significantly shorter OS and PFS, respectively. In fact, in the multivariable analysis, the high IL-15 level increased the risk of death by three times. Although the sample size was small and more studies are needed to confirm our results, our study reveals promising markers of responses to ICIs.

## 1. Introduction

Lung cancer (LC) has traditionally been considered a poorly immunogenic tumor. Currently, we know that it is one of the tumors with the highest mutational burden (MBT), with a median of approximately 10 mutations per megabase [1]. The development of immune checkpoint inhibitors (ICIs) has spurred a change in the therapeutic paradigm of LC, becoming the standard of treatment for previously untreated advanced non-small cell lung cancer (NSCLC) without actionable mutations, achieving durable responses and resulting in significant survival benefits.

In LC, an immunoresistance mechanism adopted by tumor cells is the expression of immuno-inhibitory molecules in the tumor microenvironment such as PD-L1 and CTLA-4 [2]. Therefore, using monoclonal antibodies directed against these molecules to block their action and re-establish T cell-mediated antitumor immunity has become an effective therapeutic option [3,4].

Despite the current success of ICIs, not all patients will benefit from this therapeutic strategy. Up to 50% develop resistance to treatment, a complex and dynamic mechanism in which alterations in the antigenic processing and presentation machinery, epigenetic modifications, alterations in signaling pathways (MAPK, PI3K, WNT) and the modulation of the tumor microenvironment towards a tolerogenic state can arise [5,6]. Due to this heterogeneity in responses, the need arises to identify predictive biomarkers of responses to ICIs that allow us to select which patients will benefit and which will not.

A tumor’s molecular and phenotypic characteristics are altered and modified throughout the disease and depending on the treatment, so it would be ideal to find biomarkers that reflect the changes in tumor characteristics and help us identify which tumors will respond to treatment with immunotherapy [7]. In this context, biomarkers, such as TMB and PD-L1 expression, have emerged, albeit with inconclusive results. In the search to find markers of response, the determination of the T cell receptor repertoire (TCRβ) in peripheral blood has emerged as a new predictive biomarker of the response to IT [8,9,10,11].

TCR is the antigen-specific receptor essential for the specific immune response located on the cell surface of helper and cytotoxic T lymphocytes [11]. TCR is a complex formed by two variant chains linked by disulfide bonds forming a heterodimer (αβ or γδ) that gives it the unique specificity for the antigen. Each chain has a variable (V) and a constant (C) immunoglobulin-type domain. The complementarity-determining regions (CDRs) are part of the variable chains in the TCR and are crucial for the diversity of antigen specificity generated by lymphocytes. For each chain, there are three CDRs; CDR3s have the highest variability and are encoded by the combination between V(D)J regions and are the primary sites of antigen contact. The CDR3 region of the β-chain accounts for most of the variation [11].

Statistically derived descriptive indexes have emerged to estimate the repertoire diversity and homology of TCR. TCR richness refers to the number of clonotypes that comprise the repertoire defined by the number of unique CDR3 TCRβ sequences. Clonal diversity is determined by the Shannon index, which identifies the proportions of the repertoire containing an expanded clone. Evenness is known as Shannon’s normalized diversity. It measures clonotype size similarity and ranges from 0 (the sample contains clonotypes of non-equivalent sizes as occurs in clonal expansion) to 1 (the sample is composed of clonotypes that are at the same frequency). TCR convergence refers to the frequency of identical clonotypes in amino acid sequences but different in nucleotide sequences due to codon degeneration.

TCR richness and convergence could be predictive markers of responses to ICIs. A high T cell richness and convergence level before initiating treatment with ICIs in NSCLC patients has been described as a predictive marker of response to ICIs [12]. It has also been observed that an increase in TCRβ richness and convergence during treatment with ICIs is associated with better outcomes [13].

Tumors produce a wide variety of neoantigens presented by MHC molecules and recognized by specific TCRs via TCR/peptide/MHC interactions. These T cell clones with specificity for each respective neoantigen can be reactivated again with ICIs, thereby augmenting the host antitumor immune response. Therefore, determining the TCR repertoire could allow a more precise approach and could be a more selective and effective biomarker than TMB and PD-L1 [14,15].

In contrast to TMB, TCR convergence is able to detect T cell responses to any antigen, including neoantigens arising not only from nonsynonymous mutations but also aberrant posttranslational modifications, ectopic gene expression, splicing defects, self-antigens and virus-derived antigens [16,17,18].

Determining the TCR repertoire during treatment may be a helpful tool to establish the evolution and prognosis of patients with LC. The combination of these features together with other established biomarkers, such as PD-L1 expression, would improve response prediction [17]. Even so, both TMB and TCR present certain limitations in their determination, such as the lack of standardization in the cut-off points used, and the cost of the sequencing techniques employed.

Thus, our study aims to prospectively analyze a cohort of patients with LC receiving treatment with ICIs to determine whether the TCRβ repertoire and certain soluble factors in peripheral blood involved in the regulation of T cell antitumor function such as IL-2, IL-4, IL-10, IL-12, IL-15, MICA, MICB, ULBP1, ULBP2, ULBP4, IFN-γ and CXCL10 could predict the efficacy of treatment with ICIs.

## 2. Materials and Methods

### 2.1. Population

A prospective observational study was performed on a 55-patient cohort with locally advanced and metastatic non-small cell lung cancer (NSCLC) (stage III and IV) who received treatment with PD1/PD-L1/CTLA-4 ICIs at Hospital Clínico Universitario Lozano Blesa, a tertiary hospital in Zaragoza (Spain) from April 2019 to October 2020.

Patients with histology other than NSCLC, those with contraindications for treatment with ICIs such as autoimmune diseases, synchronous tumors of other origins, patients with immunodeficiencies or at grade 2 or higher in the Eastern Cooperative Oncology Group (ECOG) scale were excluded from the study.

Patients previously treated with immunotherapy were excluded from the study. A total of 23 of 55 patients included had previously received chemotherapy, 14 patients had received treatment of localized disease (chemotherapy + radiotherapy) and 18 patients were treatment naïve.

The demographic and clinicopathologic characteristics of the patients were collected from anonymized medical records and by direct interview with the patient. The functional status of the patients was assessed using the ECOG scale. The responsible physician carried out treatment indications. Response to treatment was evaluated according to the criteria for response evaluation in solid tumors (RECIST) carried out by radiodiagnosis. The prognostic indicator variables to predict the benefit to ICIs were progression-free survival time (PFS) and overall survival (OS), defined as the time elapsed from the start of treatment to the date of disease progression or death, and to death, respectively. For those patients who did not experience progression or death, the last recorded assessment was considered the end of the study (1 October 2021).

The treatment of personal data corresponded to the Biobank of the Aragon Health System (integrated into the Spanish National Biobanks Network (PT20/00112)) after informed consent had been given and signed by the patients included in the study. The Clinical Research Ethics Committee of Aragón (CEICA) approved and evaluated the study with code (CI PI19/052). All research was performed in accordance with relevant guidelines/regulations and with the Declaration of Helsinki.

### 2.2. Sample Type and Processing

Plasma from patients was collected at baseline before starting any treatment with immunotherapy alone or immunotherapy combined with chemotherapy. Patient samples were incorporated as a final destination in the Biobank of the Aragon Health System. The analyses were performed at the Instituto de Investigación Sanitaria de Aragón (IIS Aragón) with the technological infrastructure available at the Service of Functional Genomics (SAI, Unizar/IACS) at the Biomedical Research Center of Aragon (CIBA).

#### 2.2.1. Sample Processing

The 55 peripheral blood samples were collected in tubes containing ethylenediaminetetraacetic acid (EDTA) to prevent coagulation and centrifuged at room temperature for 10 min at 2600 revolutions per minute (rpm) to separate the cells from the plasma. Subsequently, peripheral blood mononuclear cells (PBMCs) were isolated using a density gradient medium (Ficoll-Paque Plus) and incubated in RNAlater^®^ solution (Invitrogen, Carlsbad, CA, USA) overnight at 4 °C and then stored at −80 °C until processing.

A kit (MagMAX mirVana Total RNA Isolation Kit (Thermo Fisher Scientific, Waltham, MA, USA)) was used for RNA extraction from the lymphocytes isolated. The quantification, quality and measurement of the integrity of the extracted RNA were evaluated using the Qubit fluorometer, and the integrity of the extracted RNA was evaluated using the TapeStation 2200 bioanalyzer. Complementary DNA (cDNA) synthesis was carried out using the SuperScript (IV) VILO cDNA Synthesis kit (Thermo Fisher Scientific).

#### 2.2.2. Sequencing and Analysis of the TCRβ Repertoire

For sequencing, we used the Oncomine TCR Beta-SR Assay system (Thermo Fisher Scientific) targeting the CDR3 region of the TCR B-chain responsible for antigen recognition, allowing us to identify a clonotype of T lymphocytes with the same TCR (same VDJ rearrangement). For each clonotype, the nucleotide sequence of the CDR3 region (CDR3NT), the corresponding amino acid sequence (CDR3AA), and the V (variable) and J (junction) segments that compose it were reported. This platform thus allows the identification of rare or abundant clones and enables TCR convergence profiling that can measure tumor immunogenicity. The productive sequences obtained from each library were used to determine the indices that characterize the repertoire: richness, Shannon diversity, evenness and convergence.

#### 2.2.3. Determination of Cytokines in Peripheral Blood

Luminex^®^ Discovery Assay (R&D Systems a bio-techne brand) was run according to the manufacturer’s instructions in plasma, using a human premixed multi-analyte kit cytokine panel. The next soluble factors were included (IL-2, IL-4, IL-10, IL-12, IL-15, MICA, MICB, ULBP1, ULBP2, ULBP4, IFN-γ and CXCL10). Briefly, supernatants were mixed with beads coated with capture antibodies, incubated, washed and incubated with biotin-labeled detection antibodies, followed by a final incubation with streptavidin-PE. Assay plates were measured using a Luminex 200 instrument (ThermoFisher, catalog no. APX10031). Data acquisition and analysis were performed using 57 xPONENT software. The standard curve for each analyte had a five-parameter R2 value > 0.95 with or without minor fitting using xPONENT software. These determinations were carried out in collaboration with the National Centre of Oncological Investigation (CNIO) in Madrid.

### 2.3. Statistical Analysis

The population included in the study was 50 patients plus an additional 10% (5 patients) to account for possible losses, resulting in a total of 55 patients. Qualitative variables were expressed as percentages, and quantitative variables as median and standard deviation. The Kolmogorov–Smirnov test was used to test the normal distribution of a variable. The T-Student U or Mann–Whitney U test was used for independent samples according to whether the variable followed the normal distribution or not, respectively. Spearman’s correlation analysis was used to examine the association between two quantitative variables that did not follow the normal distribution (and Pearson’s correlation analysis was used if they did). For the survival analysis (OS and PFS), the nonparametric Kaplan–Meier estimator and the Mantel–Cox test were used to determine statistical significance in the comparative analysis. The Cox proportional hazard model was used on the variables detected as significant with Kaplan–Meier to determine the Hazard ratio (HR) and 95% confidence interval (95%CI).

All statistical analyses were performed using Statistical Package for the Social Sciences (SPSS) software version 24.0. Two-sided *p*-values < 0.05 were considered statistically significant.

## 3. Results

### 3.1. Descriptive Analysis

#### Patient and Tumor Disease Characteristics

The cohort of patients in our study was the same as that included in another recently published study, in which other different variables (the frequency of peripheral blood T and NK cell subsets) were analyzed [18]. A total of 55 patients were included in the study with a mean age of 65.02 years, 70.9% were male, and 98.2% were Caucasian. The vast majority (96.4%) were smokers or ex-smokers, with an Eastern Cooperative Oncology Group (ECOG) of 0 (65.5%) and no concomitant chronic infections (90.9%). Of the patients, 60% had lung adenocarcinoma, and 40% had squamous cell lung cancer. A total of 70.9% of the patients were classified with stage IV lung cancer, and only 29.1% had stage III.

PDL1 determination was performed in 47 patient tumor samples, 34% had high PDL1 expression ≥50%, 44.7% had PDL1 expression from 1 to 49% and 21.3% had an expression of PDL1 < 1%. A total of 63.6% had a baseline blood lactate dehydrogenase (LDH) level (≤214 U/L). An intermediate prognostic index LIPI score calculated by LDH and derived neutrophil/lymphocyte ratio (derived neutrophil/lymphocyte ratio ≥ 3 or LDH ≥ ULN) was present in 49.1% or poor (neutrophil/lymphocyte ratio ≥ 3 + LDH ≥ ULN) in another 49.1%.

The main indication for treatment with ICIs was being palliative in successive lines (41.8%) followed by being palliative first line (32.75%) and locally advanced (25.5%). Pembrolizumab (38.2%) was the ICI most frequently administered followed by Atezolizumab (32.7%), Durvalumab (25.5%) and Nivolumab (3.6%).

Regarding response to ICIs, 18.2% [10] experienced complete response (CR), 23.6% [13] experienced partial response (PR) and 21.8% [12] experienced disease stabilization (DS). The average time to response to ICIs was 2.74 months (95% CI 1.85–3.63), with a response duration of 8.06 months (95% CI 4.54–11.58). Despite the initial response, 56.4% (31 patients) of patients experienced disease progression.

A total of 45.5% [19] of patients presented immune-mediated toxicity: cutaneous (10.9%) and pneumonitis (10.9%) followed by endocrine (9.1%), musculoskeletal (9.1%), renal (7.3%), hepatic (5.5%) and colitis (3.6%). Immune-mediated adverse events occurred early, within the first 3 months, for endocrine, neurologic, skin and cardiovascular toxicities and late (>3 months) for the rest. Most toxicities were grade 1 and 2, and there was only one case of liver toxicity that was grade 4 (Table 1).

### 3.2. Survival Analysis

#### 3.2.1. Clinical Pathological Features

The average PFS to ICI therapy was 6.42 months (95% CI 3.97–8.87). Thirty-two patients (58.2%) had died at the time of analysis, and the time to death had a mean of 8.38 months (95% CI 5.61–11.14). The OS median was 19 months. No statistically significant differences were found in OS according to age, sex, race, smoking, BMI, latent infections, histology, immunoreactive toxicity, degree of toxicity, or PDL1 expression level. Statistically significant differences were found in OS according to ECOG (ECOG0, ECOG1), disease stage, treatment indication, the type of ICIs, the administration of corticosteroids, LIPI score, and LDH levels. (Table 2).

Regarding PFS, a statistically significant association was observed between PFS and immunorelated toxicity and tumor stage (Table 2).

#### 3.2.2. TCRβ Repertoire

##### TCRβ Sequencing Data Analysis

TCR analysis was performed on cell populations from peripheral blood samples, before starting treatment with immunotherapy. TCRβ analysis and CDR3 sequencing were finally performed on 44 samples. A total of 11 samples could not be used due to insufficient quantity or low RNA quality. The mean RNA concentration was 51.8 ng/μL, and their integrity (RIN values) had a mean of 5.2. The finally sequenced libraries had an average depth of 878,956.64 reads with an average read length of 88 base pairs per library. From the total number of libraries, three were discarded due to low quality, and the rest had 50% or more productive reads, indicating that the sequencing of all samples was satisfactory and sequencing analysis could continue.

**Figure 1:**
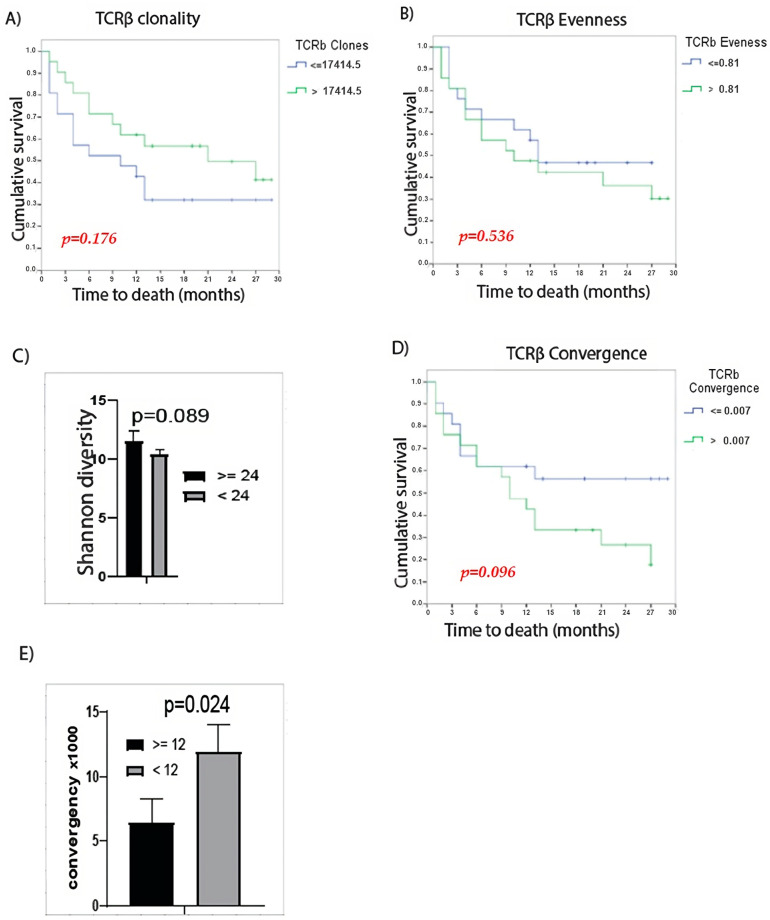
Characteristics of the TCRβ repertoire and its relationship with OS or PFS. (**A**) Kaplan–Meier curves for OS of patients with TCRβ clonality that was high vs. low (*p* = 0.176), (**B**) Kaplan–Meier curves for OS of patients with TCRβ evenness that was low vs. high (*p* = 0.536), (**C**) TCRβ Shannon diversity between patients with OS < or ≥24 months, (**D**) Kaplan–Meier curves for OS of patients with TCRβ convergence that was high vs. low (*p* = 0.096), (**E**) TCRβ convergence between patients with PFS < or ≥12 months. Mantel–Cox test was used to determine statistical significance.

The productive sequences obtained from each library were used to determine the indices that characterize the TCRβ repertoire, as explained in the methodology. The mean of TCRβ evenness was 0.77 ± 0.14 (median: 0.81 ± 0.14), the Shannon diversity index was 10.7 ± 2.56 (median: 10.9 ± 2.56) and the TCRβ convergence pre-treatment was 0.01 ± 0.01 (median: 0.007 ± 0.01). The median of the different TCRβ variables was chosen to divide the cohort into two groups. The cohort was divided into two groups for each variable according to the median to analyze the impact of TCRβ repertoire characteristics on OS and PFS (Figure 1).

No statistically significant differences were observed in OS or PFS according to TCRβ clonality (Figure 1A) and TCRβ evenness (Figure 1B). However, there was a tendency to increase the OS and PFS with higher clonality and, therefore, a better ICI response. Regarding TCRβ Shannon diversity, those patients with a survival ≥24 months had higher levels of TCRβ Shannon diversity with a trend to statistical significance (*p* = 0.089) (Figure 1C). Finally, when analyzing TCRβ convergence, there were no differences in OS (*p* = 0.096) nor in PFS between the TCRβ convergence groups, although there was a trend that at a lower convergence, OS was higher (Figure 1D). When comparing TCRβ convergence between patients with PFS < or ≥ 12 months, statistically significant differences were observed with lower convergence in patients with PFS ≥ 12 months (Figure 1E).

#### 3.2.3. Cytokines and Other Soluble Factors

##### Analysis of the Determination of Cytokines

The levels of 10 cytokines in 54 pre-treatment peripheral blood patient samples were analyzed (Table 3).

**Figure 2:**
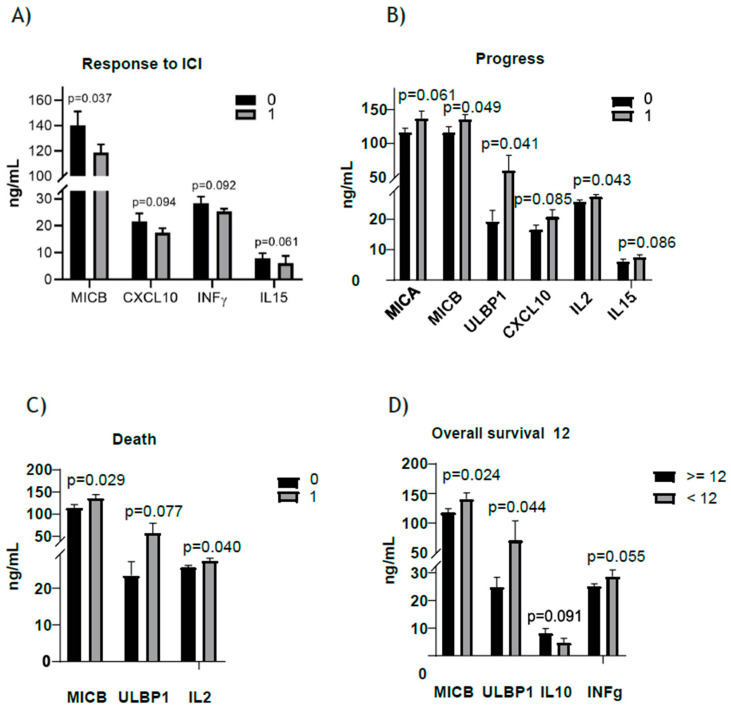
Analysis of expression of cytokines. (**A**) Levels of cytokines between ICI responders (=1) and non-responders (=0), (**B**) Levels of cytokines between patients with disease in progression (=1) and no progression (=0), (**C**) Levels of cytokines between dead patients (=1) and not dead patients (=0), and (**D**) Levels of cytokines in those with overall survival ≥12 months.

An analysis was performed between ICI responders and non-responders, selecting only those cytokines or soluble factors that were statistically significant or close to significance such as MICB, CXCL10, IFNγ and IL15 (Table 4). Responders showed lower MICB values before initiating therapy, reaching statistical significance (*p* = 0.037) (Figure 2).

Patients with no disease progression also had significantly lower levels of MICB (*p* = 0.049), ULBP1 (*p* = 0.041) and IL2 (*p* = 0.043). Likewise, those who had not died at the end of the analysis, showed lower levels of MICB (*p* = 0.029) and IL-2 (*p* = 0.04), and those who survived 12 months or more had statistically significantly lower levels of MICB (*p* = 0.024) and ULBP1 (*p* = 0.044) (Figure 2).

**Figure 3:**
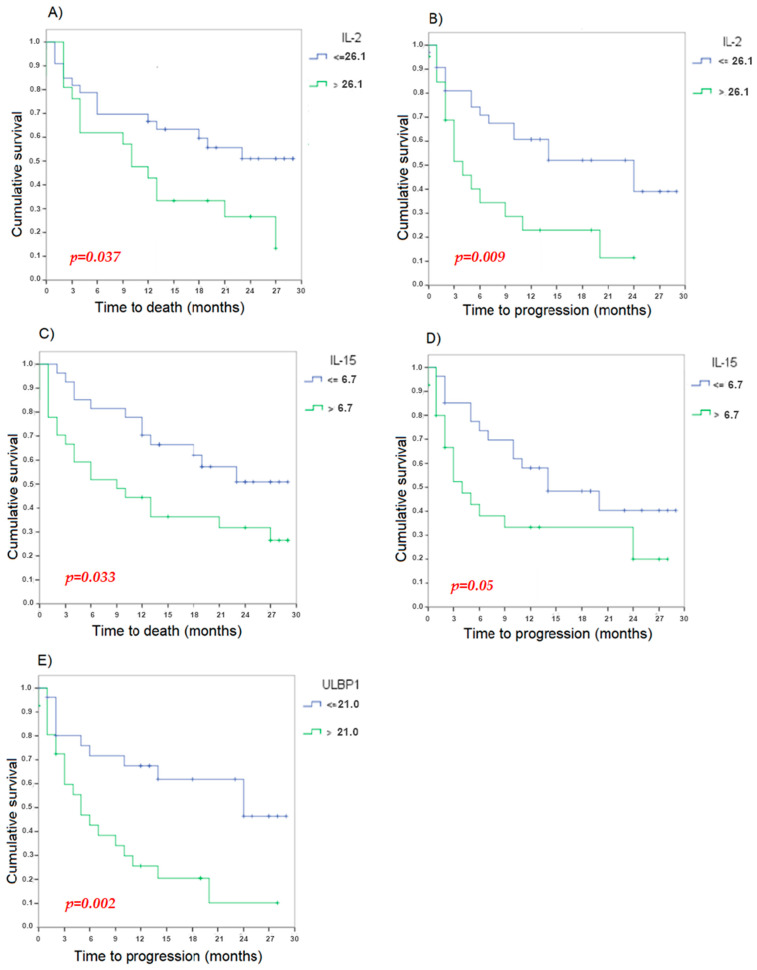
Characteristics of cytokines and their relationships with overall survival and progression-free survival. (**A**) Kaplan–Meier curves for OS of patients with levels of IL-2 >26.1 vs. levels of IL-2 ≤26.1 (*p* = 0.037), (**B**) Kaplan–Meier curves for PFS of patients with levels of IL-2 > 26.1 vs. levels of IL-2 ≤ 26.1 (*p* = 0.009), (**C**) Kaplan–Meier curves for OS of patients with levels of IL-15 > 6.7 vs. levels of IL-15 ≤ 6.7 (*p* = 0.033), (**D**) Kaplan–Meier curves for PFS of patients with levels of IL-15 > 6.7 vs. levels of IL-15 ≤ 6.7 (*p* = 0.050), and (**E**) Kaplan–Meier curves for PFS of patients with levels of ULBP1 >> 21.0 vs. levels of ULBP ≤ 21.0 (*p* = 0.002).

A second analysis was carried out. The cohort was divided for each soluble factor using the median to analyze the impact of each one on OS and PFS. It was observed that those patients with levels of IL-2 > 26.1 presented shorter OS (*p* = 0.037) and shorter PFS (*p* = 0.009). Similarly, those patients with levels of IL-15 > 6.7 had shorter OS (*p* = 0.033) and PFS (*p* = 0.050). Significant differences were observed in PFS between groups according to ULBP1 levels (*p* = 0.002), with PFS being lower if the levels of ULBP1 > 21.0. Although not statistically significant (*p* = 0.058), it was observed that those patients with levels of IL-10 > 2.8 tended to have higher OS (Figure 3). Regarding the rest of the cytokines: IL-4, IL-12, MICA, MICB, ULBP2, ULBP4, IFN-γ and CXCL10 no statistically significant differences were observed between expression level and OS or PFS (Figure 3).

### 3.3. Multivariate Analysis

In order to develop an analysis for identifying independent predictive biomarkers, we performed a multivariate analysis. We studied the relationship between variables with *p*-values < 0.05 in the univariate analysis and OS. For this purpose, the Cox regression model was used. The variables included were ECOG, tumor stage, the indication for treatment, the type of ICI, the best response, LDH, IL-2 and IL-15. The variables of immune-related toxicity and IL-10 were also added, as they were close to being significant. The coefficient estimate, standard error, significance, hazard ratio and confidence interval are shown in Supplementary Table S1 as the hazard ratio (log HR) confidence interval for each variable (Supplementary Table S1).

The multivariate COX analyses (Table 5) showed the simultaneous analysis of more than one response variable according to the Cox analysis. Some variables appeared to be associated with OS. For the same reason, these variables validate our statistical model and the use of IL-15 as a biomarker to predict the ICI therapy response. As can be seen, patients with high IL-15 levels in plasma had a shorter OS, indicating that IL-15 is an independent prognostic factor. Elevated IL-15 levels in plasma increased the risk of death by three times (HR = 3, 95% CI: 1.368–6.578, *p* < 0.006).

In the same multivariate model, other variables showed a statistical correlation. Patients with an indication for palliative first-line treatment had a 10.63 times higher risk of death than those with locally advanced treatment. On the other hand, patients with palliative successive lines of treatment had a 15-fold increased risk of death. The ICI response variable showed great variability (possibly due to the scarcity of data within each category). Patients with PD increased the risk of death 53.256 times over CR patients. ECOG1 patients had 2.5 times the risk of death over ECOG0, in any time unit and adjusted for all other confounding variables. Those patients with stage IV tumor disease had a 10-fold higher risk of death than stage III patients.

**Figure 4:**
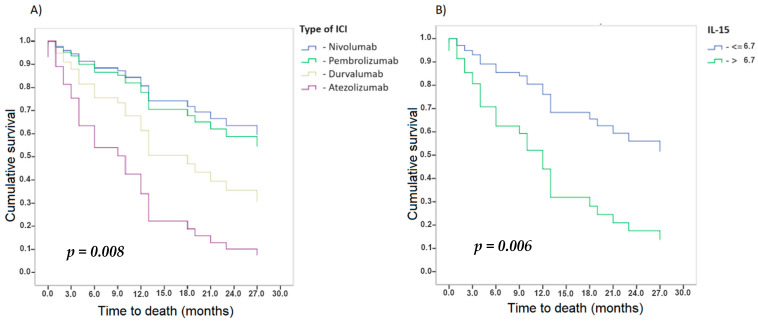
Kaplan–Meier curves of survival functions for the different levels of the influential categorical factors. (**A**) OS according to type of ICI, (**B**) OS according to IL-15.

In previous studies, all these variables have already been demonstrated to affect OS and are used in clinical practice. Hence, they validate our model and the use of IL-15 as a biomarker to predict the ICI therapy response. The following graphs show the survival functions estimated with the proposed model for the different levels of the influential categorical factors (Figure 4).

## 4. Discussion

Although immunotherapy has revolutionized lung cancer management, we still need to identify markers to help us better select patients who will benefit from it, thus avoiding toxic treatments and a lack of therapeutic benefit in non-responders.

With this objective, we designed a study that included a cohort of patients with advanced and locally advanced lung cancer in which the characteristics of the TCRβ repertoire in mononuclear cells and certain soluble factors were analyzed in peripheral blood samples before the initiation of ICIs in monotherapy or in combination with chemotherapy. Although our study’s sample size is limited, the cohort included is representative of the lung cancer population, which may help us draw certain conclusions in this regard.

There is evidence that the analysis of the TCRβ profile provides predictive information on responses to ICIs [8]. However, the evidence in this regard is quite variable, possibly due to small sample sizes, studies performed in different tumor pathologies, the type of immunotherapeutic agent administered and the sequencing methods performed [20]. One of the advantages of using TCRβ as a biomarker is that it can be determined in an easily accessible sample, such as peripheral blood, without resorting to tumor tissue, which is often scarce in lung cancer [21]. Evaluating TCR clonality in both compartments, blood and tissue, can provide valuable insights into the dynamics of the immune response in lung cancer patients. In lung cancer, it has been observed that a more clonal TCR repertoire in tumor tissue is associated with greater T cell infiltration and a better response to immunotherapy. However, the exact relationship between clonality in peripheral blood and tumor tissue may vary, as peripheral blood does not always reflect the clonal diversity present in the tumor microenvironment [19]. Even so, TCRβ has certain limitations regarding its determination, such as the lack of standardization in the cut-off points, the platforms and the cost of the sequencing techniques employed.

In our study, it was observed that there is a trend, although not significant, that the greater the richness or baseline number of different TCRβ clonotypes, the greater the clinical benefit, achieving an increase in OS. Previous studies have shown that treatment with ICIs can have a pharmacodynamic effect by increasing the number of unique TCR clonotypes in peripheral blood and, consequently, a greater possibility of recognizing tumor neoantigens, thus improving therapeutic response [14,19]. This has been proven by analyzing tumor tissues in responder patients with NSCLC receiving treatment with neoadjuvant ICIs, observing tumor tissue enriched with expanded clonotypes [14]. Thus, it has been observed that the pre-treatment presence of clones in the tumor shows clonal expansion in blood after treatment, correlating with better response and the clearance of circulating tumor DNA [20].

TCRβ diversity refers to the number of clonotypes present. It is to be expected that the greater the diversity of the TCRβ repertoire, the greater the probability that T lymphocytes will recognize tumor antigens and, therefore, have the best antitumor response. Han, J. et al. 2020 and Huang, A.C. et al. 2017 showed that the diversity of the PD-1+ CD8+ TCR population in blood could indicate that there is a greater proportion of exhausted T cells that can be reactivated with ICIs, leading to a more effective immune response in patients with non-small cell lung cancer [22,23]. However, the conclusions reached by previous studies regarding TCRβ diversity are pretty mixed. In fact, in our study, no significant differences in OS or PFS were observed according to the TCRβ Shannon diversity index. However, there was a trend towards better OS after treatment in those with greater TCRβ Shannon diversity. This can be explained by the fact that there are antigen-specific TCRs in the peripheral blood mononuclear cells that are non-tumorigenic and can dilute tumor-specific TCRs [19].

Therefore, our study confirms that a wide repertoire of TCR in blood with greater richness and diversity makes the recognition of tumor antigens more likely and that they are reactivated later with the action of ICIs, thus decreasing the immune escape of tumor cells.

In terms of the convergence analysis, it has been seen that patients with a greater response to ICIs have a greater pre-treatment TCR convergence, thus reinforcing the idea that T cells with convergent TCRs target tumor antigens [16]. An advantage of TCR convergence as a biomarker is that it is able to detect the T cell response to tumor neoantigens beyond those originating from nonsynonymous mutations (point mutations that alter the resulting protein sequences). The evidence in this regard is contradictory to what was observed in our study, in which no significant differences in OS or PFS were observed according to TCRβ convergence, and even a tendency was observed that the lower the TCRβ convergence, the higher the OS and PFS. TCR convergence is a process by which a tumor antigen determined by antigenic specificity leads to the expansion of T cells that share TCRs with antigen specificity. Furthermore, some studies suggest that successful immunotherapy was not reliant on the select expansion of specific T cell clones, but instead induced the relatively uniform expansion of most tumor-infiltrating T cells, enhancing effector capabilities [24]. However, different therapeutic approaches and distinct cancers could likely yield different results.

It has also been shown that the sequencing method or platform chosen for analysis can vary in determining TCRβ [16]. Thus, a study comparing TCRβ sequencing using the Illumina platform with the Oncomine assay was performed and differences were seen between them. Both were consistent in detecting TCR clonality and diversity, but Illumina resulted in a higher detection of convergent TCRs [16]. With prior knowledge of the different substitution error rates in the different sequencing platforms, the most appropriate platform can be chosen accordingly.

In our study, the Oncomine platform was used. Perhaps the sequencing platform employed with a lower detection rate than others, such as Illumina along with the paucity of peripheral blood samples that could be used for TCRβ determination, contributed to these results. Still, further understanding of the mechanisms involved and studies involving larger cohorts are required to consider TCR as a predictive biomarker of responses to ICIs.

It has been shown that several factors in peripheral blood prior to initiating treatment with ICIs, such as the number of activated CD4 memory T cells and a more clonally diverse TCR repertoire, are associated with the development of severe immune-mediated adverse effects and with a greater response to ICIs [25]. In addition, it has been observed that patients receiving treatment with ICIs experience changes in TCR clonality that may be related to the severity of the immune-mediated event and the timing of the event [25]. In fact, according to other studies, patients with NSCLC with greater increases in PD-1+ CD8+ TCR repertoire clonality intra and post-ICI presented greater PFS and OS, reflecting an expansion of a successful anti-tumoral clonotype. On the contrary, pre-treatment TCR repertoire diversity could be a treatment-agnostic prognostic factor [26].

These findings could be of great utility because the modification in the characteristics of the TCRβ repertoire during treatment could serve as a tool to predict and identify which patients are at a higher risk of developing them. In our cohort, it was seen that patients with higher toxicity had higher values of TCRβ convergence and as mentioned, this has been found to be associated with higher response to ICIs.

A proinflammatory gene expression profile in pre-treatment samples is associated with a superior pathologic response after treatment with chemotherapy and immunotherapy [27]. Thus, pre-treatment peripheral blood analysis of some cytokines was performed to study whether certain pre-treatment blood soluble factors could predict the response to or benefit of ICIs.

In our cohort, it was observed that higher pre-treatment levels of IL-2 and IL-15 were associated with more aggressive tumor behavior and worse outcomes: patients survived less and had lower PFS. This is consistent with evidence from other studies [28]. In patients with lung cancer, high concentrations of intratumoral IL-15 are associated with a worse prognosis [29]. It appears that intratumoral production and/or circulating sIL-15/IL-15Rα complexes contribute to developing a tumor microenvironment favorable for tumor progression and immune escape [30]. However, there are exceptions to the behavior described above since, in some solid tumors, the IL-15/IL-15R complex may also play an antineoplastic role [29,30]. Therefore, the role of intratumoral and circulating IL-15 is complex and depends on several factors, such as the type of IL-15 produced, the IL-15-Rα chain isoforms involved in sIL-15/IL-15Rα, the presence of functional IL-15 receptors on tumor cells, as well as their response to stromal and endogenous IL-15. Therefore, it is difficult to say whether it predicts aggressive behavior [30]. Although there is no data regarding levels of circulating IL-15 in relation with antiPD1/PDL1 efficacy, a previous study showed that low serum IL-15 levels correlates with better responses to antiCTLA4 treatment in melanoma [31].

Regarding IL-2, this cytokine seems to have a dual effect on the tumor immune microenvironment. On the one hand, ICIs could, by interacting with T cells, increase IL-2 secretion, enhancing the immune response, but recent studies show that IL-2 also induces immunosuppressive activity by promoting Treg proliferation and activation, which inhibits the antitumor response [32]. Therefore, further studies are needed to investigate and clarify the relationship between IL-2 and ICIs.

No statistically significant association was observed in the univariate analysis between IL-10 levels and OS. IL-10 is an immunosuppressive and anti-inflammatory cytokine that regulates the growth and differentiation of different cell types. It is well known that in cancer patients, higher levels of IL-10 in serum correlate inversely with oncologic prognosis [32,33]. Despite this, it has recently been observed that IL-10 may play a role in CD8+ T cell activation and proliferation in cancer and chronic inflammation [32]. In addition, IL-10 and PD-1 play immunosuppressive roles through very different pathways [33], and a dual blockade has synergistic antitumor action [34,35,36]. Their efficacy and safety as antitumor therapy has been proven in several studies [37]. Considering the heterogeneity of the findings regarding this cytokine, the small size of our sample, and the arbitrary value taken to consider high IL-10 expression (>2.8), more studies and data are needed to clarify the prognostic significance of IL-10 in the treatment of ICIs.

IFN-γ exerts a dual role. On the one hand, it is a potent inducer of the antitumor immune response, but it can also serve as a tumor escape mechanism. Its direction towards one or the other action will depend on tumor specificity, signal intensity and the tumor microenvironment [38]. In our work, we found no association between an IFN-γ expression signature and a response to ICIs; in fact, responders were observed to have lower levels of MICB, CXCL10 and IFN-γ. Several studies have shown that ICIs increase IFN-γ production, contributing to tumor clearance, and it has been demonstrated that resistance to IT could be due to defects in the IFN-γ signaling pathway [38,39]. The IFN-γ and PD-L1 gene signature combination has been associated with a greater therapeutic benefit to IT and could constitute a predictive biomarker of response to ICIs [40,41,42,43].

It has been shown in different studies that tumors with higher CXCL10 expression correlate with a better prognosis [44]. On the contrary, in our cohort, it was seen that responder patients had lower pre-treatment levels of CXCL10. This soluble factor is involved in T and NK cell mobilization. Thus, patients with low levels of this factor may have more of a CXCL10 increase after ICI treatment and, therefore, a better response. Deep research is needed to confirm this hypothesis.

The important role of NK cells and NKG2D ligands in cancer immunosurveillance suggests that their presence in serum could serve as a prognostic marker [45]. Their relationship with survival in cancer patients has been studied [46,47,48,49]. The ligands of NKG2D are MICA, MICB and six members of the ULBP family [50]. The release of soluble NKG2D ligands represents a form of tumor cell immune evasion strategy since these ligands deregulate NKG2D expression by decreasing NK cell function and T cell activation, and furthermore these soluble ligands compete in receptor binding with ligands expressed on the surface of tumor cells [45]. Therefore, it is to be expected that higher levels are associated with worse prognosis and disease progression, although its relation with ICI response has not been previously determined.

These findings are consistent with our work, in which patients with lower levels of ULBP1, ULBP2, MICB and MICA had better survival outcomes. Higher levels of ULBP1 were significantly associated with lower PFS. Patients with OS ≥ 12 months had statistically lower levels of MICB, ULBP1 and ULBP4. Although these ligands may play an essential role as predictors of the evolutionary course of cancer, the complex regulation of NKG2D ligands, their variation, and specificity depending on tumor type will have to be taken into account, and a better future understanding of the effects of these soluble factors on immune cells will be necessary [51].

The difference in cytokine serum level values between responders and non-responders could be appreciated as small. However, serum determination is an indicator of the different levels in the tumor microenvironment (TME), meaning the differences could be higher in the TME. Other publications report similar or minor differences between healthy and patient serum samples and between treated and non-treated patients with statistical differences and biological significance [52]. In our research, serum determinations were performed in all patients before starting treatment, so it is normal not to see large differences. However, these slight variations seem to help predict the response to ICIs. Of course, these findings need to be corroborated by subsequent studies using a bigger cohort of patients, but if so, they would be a valuable tool in clinical practice. Other publications in pediatric autoimmune hepatitis also detected minor differences between groups and showed how IL-2 levels predicted treatment response [53]. The relevance of our research just lies in the relatively few studies that have been published describing the relationship between cytokines and ICI responses.

The differences (or trends) in PFS and OS associated with TCR diversity and cytokines might be indicating a prognostic role of these biomarkers but there is not enough evidence to confirm the predictive role of the proposed biomarkers.

The cohort included in the study was heterogeneous, as it included patients with both localized and metastatic lung cancer, who therefore had different tumor burdens. Moreover, it is well established that the efficacy and possibly the underlying molecular mechanisms of immune checkpoint inhibitors (ICIs) in patients receiving immunotherapy after progression on platinum-based chemotherapy is different from untreated patients. To verify that the predictive value of cytokines and TCR diversity as markers of response to immunotherapy is not influenced by tumor burden among patients with localized and advanced lung cancer, we conducted a comparison using a *t*-test (Supplementary Materials Table S2). As shown, no significant differences were observed between the two groups, indicating that the results are not influenced by tumor burden. Although larger cohorts would be necessary to confirm these results, we suggest that the expression levels of certain cytokines and TCR diversity may have a predictive value for immunotherapy response.

In summary, we found in our study that the high clonality and diversity of the TCR repertoire and low levels of IL-2 and IL-15 are associated with a greater response to immunotherapy. This relationship between lower levels of interleukins and a greater response to immunotherapy treatment may seem paradoxical and contradictory, as both cytokines are known to induce the proliferation and activity of T and NK cells. This could be due to the fact that interleukins IL-2 and IL-15 play a role in the regulation and counter-regulation of the immune response. Very high levels of these cytokines can lead to an overactivation of the immune system, which may result in the induction of tolerance and immunosuppressive mechanisms [54]. On the other hand, the immune response is dynamic, and low levels of IL-2 and IL-15 may indicate a more balanced immune environment that favors an effective antitumor response. However, persistently high levels can result in a chronic inflammatory response, contributing to an immunosuppressive environment [55].

Additionally, it has been observed that ICIs can be more effective in the presence of not very high levels of IL-2 and IL-15, allowing T and NK cells to respond more effectively to the treatment.

Concerning markers predictive of immune toxicity during treatment, in our study, an association was observed between IL-15 and MICB expression and the development or not of immune-mediated toxicity; thus, those who did not present toxicity had higher levels of IL-15 and MICB and lower survival. Therefore, although more evidence is needed, it would be interesting to validate these findings in future studies and to observe the role of these soluble factors as predictors of immune toxicity and thus, of responses to ICIs.

## 5. Limitations

The small sample size is the main limitation of our study, which probably prevented us from reaching statistical significance for several of the variables studied. Another limitation is the heterogeneity of the cohort, related to the inclusion of patients with localized NSCLC and metastatic patients, which affects intrinsic characteristics of response to immunotherapy treatment.

TCRβ is a dynamic marker that can be modified throughout the disease course of a cancer patient being treated with IT. In the same way, ICIs can modify the basal characteristics of that TCRβ repertoire. In our study, the TCRβ analysis was only performed before the start of treatment. Therefore, although it may help us predict which patients may benefit most from treatment, it would have been interesting to analyze how the TCRβ repertoire is modified. Another limitation of our study is that we did not study the characteristics of the TCRβ repertoire according to the patient’s demographic characteristics and smoking habits and that these models will require validation in larger independent cohorts of LC patients.

## 6. Conclusions

Characteristics of the TCR repertoire and cytokines such as IL-2 and IL-15 constitute promising molecular markers of response to immunotherapy treatment. In addition, IL-15 appears to be involved in immune-mediated toxicity. However, future studies are needed to consolidate our results in order to apply them in clinical practice.

## Figures and Tables

**Table 1:** Overview of the patient cohort comprised in the study that includes 55 LC patients. Demographic parameters such as sex, age, gender, race, smoking habits, PD-L1, LDH, LIPI score, histology, tumor stage, treatment indication, ICI, ICI response, death, immune-mediated toxicity and type of immune-mediated toxicity are included.

Variable	Total: 55 Patients (N)	(%)
Sex		
Males	39	70.9
Female	16	29.1
Age		
Mean age: 65.02		
<5	47	85
≥75	8	15
ECOG		
ECOG 0	36	65.5
ECOG 1	19	34.5
IMC		
<30 kg/m^2^	46	83.6
≥30 kg/m^2^	9	16.4
Race		
Caucasian	54	98.2
Others	1	1.8
Smoking Habit		
Never smoker	2	3.60
Former smoker/Current smoker	53	96.40
PD-L1		
<1%	10	18.20
1–49%	21	38.20
≥50%	16	29.10
Unknown	8	14.50
LDH		
Normal (≤214 U/L)	35	63.60
High (>214 U/L)	20	36.40
LIPI Score		
Poor	27	49.10
Intermediate	27	49.10
Good	1	1.80
Histology		
Adenocarcinoma	22	40
Squamous	33	60
Tumor Stage		
III	16	29.1
IV	39	70.9
Treatment Indication		
Locally advanced	14	25.50
First line	18	32.70
Second line or more	23	41.80
ICI		
Durvalumab	14	25.50
Pembrolizumab	21	38.20
Atezolizumab	18	32.70
Nivolumab	2	3.60
ICI Response		
Complete response (CR)	10	18.80
Partial response (PR)	13	23.60
Stable disease (SD)	12	21.80
Progressive disease (PD)	15	27.30
Not evaluable (NE)	5	
Death		
Yes	32	58.20
No	23	41.80
Immune-Mediated Toxicity		
Yes	25	45.50
No	30	54.50
Immune-Mediated Toxicity		
Skin	6	24
Pneumonitis	6	24
Endocrine	5	20
Musculoskeletal	5	20
Renal	4	16
Liver	3	12
Colitis	2	8

ECOG: Eastern Cooperative Oncology Group, LDH: lactate dehydrogenase, LIPI: Lung Immune Prognostic Index.

**Table 2:** Analysis of the influence of each variable on patient survival (OS and PFS). Statistical analysis was conducted to assess the influence of each variable (sex, age, ECOG, BMI, race, smoking habit, PD-L1 expression, LDH, LIPI score, histology, tumor stage, treatment indication, ICI, ICI response, immune-mediated toxicity, type of immune-mediated toxicity) on patient survival (OS and PFS). Nonparametric Kaplan–Meier estimators and the Mantel–Cox test were utilized to determine statistical significance in the comparative analysis.

	Overall Survival	Progression-Free Survival
	IC (11.13–26.87)	IC (2.81–17.19)
	*p* value	*p* value

Sex		
Males	0.396	0.646
Female		
Age		
Mean age: 65.02	0.065	
<75		0.170
≥75		
ECOG		
ECOG 0	0.000	0.643
ECOG 1		
IMC		
<30 kg/m^2^	0.695	0.889
≥30 kg/m^2^		
Race		
Caucasian	0.196	0.113
Others		
Smoking Habit		
Never smoker	0.165	0.528
Former smoker/Current smoker		
PD-L1		
<1%	0.194	0.389
1–49%		
≥50%		
Unknown		
LDH		
Normal (≤214 U/L)	0.017	0.086
High (>214 U/L)		
LIPI Score		
Poor	0.000	0.005
Intermediate		
Good		
Histology		
Adenocarcinoma	0.487	0.713
Squamous		
Tumor Stage		
III	0.000	0.034
IV		
Treatment Indication		
Locally advanced	0.000	0.076
First line		
Second line or more		
ICI		
Durvalumab	0.000	0.354
Pembrolizumab		
Atezolizumab		
Nivolumab		
ICI Response		
Complete response (CR)	0.000	0.000
Partial response (PR)		
Stable disease (SD)		
Progressive disease (PD)		
Not evaluable (NE)		
Immune-Mediated Toxicity		
Yes	0.051	0.030
No		
Immune-Mediated Toxicity		
Skin		
Pneumonitis	0.588	0.697
Endocrine		
Musculoskeletal		
Renal		
Liver		
Colitis		

ECOG: Eastern Cooperative Oncology Group, LDH: lactate dehydrogenase, LIPI: Lung Immune Prognostic Index.

**Table 3:** Analysis of the determination of cytokines.

Cytokine	Median	Mean	Standard Deviation	Interquartile Range
MICA	119.5	128.0	49.5	44.9
MICB	117.4	126.7	42.4	49.6
ULBP1	21.0	42.7	96.7	16.1
ULBP2	157.5	159.5	22.9	27.9
CXCL10	16.4	19.0	11.0	11.1
IL10	2.8	6.7	9.2	9.9
ULBP4	0.0	27.6	81.4	20.6
IFNγ	25.5	26.5	8.0	5.1
IL4	0.0	18.0	23.8	37.3
IL2	26.1	26.7	3.5	2.9
IL15	6.7	7.0	3.8	2.8
IL12	0.0	137.7	412.2	153.9

**Table 4:** Analysis of cytokines between ICI responders and non-responders.

Cytokines	Response to ICI	N	Mean	*p* Value
MICA	No	20	133.7	0.516
	Yes	34	124.6	
MICB	No	20	140.2	0.037
	Yes	34	118.8	
CXCL10	No	20	21.6	0.094
	Yes	34	17.5	
IFNγ	No	20	28.4	0.092
	Yes	34	25.4	
ULBP1	No	20	52.1	0.589
	Yes	34	37.2	
ULBP2	No	20	162.8	0.206
	Yes	34	157.5	
IL10	No	20	4.0	0.162
	Yes	34	5.6	
ULBP4	No	20	9.2	0.302
	Yes	34	7.1	
IL4	No	20	19.6	0.356
	Yes	34	17.0	
IL2	No	20	26.3	0.319
	Yes	34	26.0	
IL15	No	20	7.4	0.061
	Yes	34	6.2	
IL12	No	20	204.6	0.183
	Yes	34	98.4	

**Table 5:** Multivariate analysis.

Covariable	Coefficient Estimate (B_i_)	SD Estimation	Sig.	Exp (B) (HR.)	IC (HR) 95%
TI Locally advanced	-	-	0.006	-	-
TI First line	2.363	0.901	0.009	10.627	1.816–62.181
TI Second line or more	2.708	0.852	0.001	14.998	2.823–79.679
ECOG [1]	0.915	0.393	0.020	2.496	1.155–5.391
Staging (IV)	2.295	1.081	0.034	9.929	1.193–82.625
IL-15 (>6.7)	1.098	0.401	0.006	3.000	1.368–6.578
Atezolizumab	-	-	0.008	-	-
Nivolumab	−1.615	1.082	0.135	0.199	0.024–1.657
Pembrolizumab	−1.457	0.444	0.001	0.233	0.098–0.557
Durvalumab	−0.790	1.277	0.536	0.454	0.037–5.540

## Data Availability

The data presented in this study are available on request from the corresponding author.

## Supplementary Materials

The following supporting information can be downloaded at: https://www.mdpi.com/article/10.3390/cancers16162798/s1, Supplementary Table S1. Individual coefficients of the variables included in the Cox regression. Supplementary Table S2. Comparison of Variables Between Localized and Advanced NSCLC Patients. To ensure that the observed differences in various variables are not attributable to tumor burden, patients with localized Non-Small Cell Lung Cancer (NSCLC) were compared to those with advanced NSCLC for each variable. As demonstrated, no statistically significant differences were observed between the two patient groups. Statistical analysis was performed using an independent samples *t*-test.
