# Supplementary material for: Peripheral Blood TCRβ Repertoire, IL15, IL2 and Soluble Ligands for NKG2D Activating Receptor Predict Efficacy of Immune Checkpoint Inhibitors in Lung Cancer
Source: Cancers (Basel). 2024 Aug 8;16(16):2798. doi: 10.3390/cancers16162798 (PMC11352724; doi:10.3390/cancers16162798)
Supplement: Supplementary file 1 [file cancers-16-02798-s001.zip › cancers-3103719-supplementary.pdf]

Supplementary data

Supplementary Table S1. Individual coefficients of the variables included in the Cox regression.

Covariates	Coefficient estimate (B_i)	SD of estimation	Sig.	Exp (B) (HR.)	IC (HR) 95%
ECOG 1	1.178	0.359	0.001	3.247	1.606-6.562
Stage (IV)	1.878	0.609	0.002	6.539	1.983-21.565
TI Locally advanced			0.001		
TI First line	1.622	0.775	0.036	5.063	1.108-23.126
TI Palliative successive lines	2.553	0.748	0.001	12.840	2.962-55.656
Type of ICI (Durvalumab)			0.001		
Type of ICI (Nivolumab)	1.205	1.225	0.325	3.336	0.302-36.831
Type of ICI (Pembrolizumab)	1.735	0.765	0.023	5.668	1.266-25.376
Type of ICI (Atezolizumab)	2.749	0.757	0.000	15.635	3.543-68.922
Complete Response (CR)			0.000		
Parcial Response (PR)	1.522	1.097	0.165	4.582	0.534-39.348
Stable Disease (SD)	2.126	1.072	0.047	8.384	1.026-68.509
Progressive disease (PD)	3.634	1.051	0.001	37.880	4.830-297.063
Not evaluable	5.933	1.240	0.000	377.216	33.191-4287.125
Immunorelated toxicity (No)	0.691	0.367	0.060	1.995	0.972-4.095
LDH (High (>214 U/L))	0.816	0.357	0.022	2.262	1.124-4.551
IL-2 (>26.1)	0.739	0.362	0.041	2.093	1.031-4.252
IL-10 (≤2.8)	0.675	0.370	0.069	1.963	0.950-4.059
IL-15 (>6.7)	0.765	0.371	0.039	2.149	1.039-4.444

ECOG: Eastern Cooperative Oncology Group; TI: Treatment indication; LDH: Lactate dehydrogenase

Supplementary Table S2. Comparison of Variables Between Localized and Advanced NSCLC Patients. To ensure that the observed differences in various variables are not attributable to tumor burden, patients with localized Non-Small Cell Lung Cancer (NSCLC) were compared to those with advanced NSCLC for each variable. As demonstrated, no statistically significant differences were observed between the two patient groups. Statistical analysis was performed using an independent samples t-test.

VARIABLE	MEAN TUMOR STAGE III	MEAN TUMOR STAGE III	P value	Significantly different?
TCRβ Shanon diversity	9,900	10,94	0,2495	ns
TCRβ Eveness	0,7235	0,7808	0,2611	ns
TCRβ Convergency x1000	8,764	10,38	0,6428	ns
MICA	123,3	129,9	0,6583	ns
MICB	111,6	133,1	0,2291	ns
ULBP1	27,52	49,06	0,4599	ns
ULBP2	158,1	160,1	0,7772	ns

CXCL10	20,50	18,35	0,5186	ns
IL10	6,939	6,606	0,9042	ns
ULBP4	6,257	36,60	0,2140	ns
IFNg	27,22	26,16	0,6609	ns
IL4	10,50	21,11	0,1365	ns
IL2	26,47	26,75	0,7935	ns
IL15	8,172	6,525	0,1476	ns
IL12	65,90	167,9	0,4115	ns
